# Hypoxia-induced circRNF13 promotes the progression and glycolysis of pancreatic cancer

**DOI:** 10.1038/s12276-022-00877-y

**Published:** 2022-11-11

**Authors:** Qiuyan Zhao, Zhonglin Zhu, Wenqin Xiao, Guanzhao Zong, Chuanyang Wang, Weiliang Jiang, Kai Li, Jie Shen, Xingya Guo, Jianhua Cui, Lihong Guo, Rong Wan

**Affiliations:** 1 Department of Gastroenterology, Shanghai General Hospital, Shanghai Jiao Tong University School of Medicine, Shanghai, China; 2 Shanghai Key Laboratory of Pancreatic Diseases, Shanghai General Hospital, Shanghai Jiao Tong University School of Medicine, Shanghai, China; 3 Department of Colorectal Surgery, Fudan University Shanghai Cancer Center, Shanghai, China; 4 Department of Gastroenterology, Dongtai People’s Hospital, Jiangsu, China; 5 Department of Gastroenterology, Shengli Oilfield Central Hospital, Shandong, China

**Keywords:** Cancer microenvironment, Non-coding RNAs

## Abstract

Pancreatic cancer (PC) is one of the most malignant tumors. Rapid progression and distant metastasis are the main causes of patient death. Hypoxia is a hallmark of multiple cancers and is involved in tumor biology. However, little is known about the roles of circRNAs in glycolysis and hypoxia-mediated progression of PC. Here, the expression pattern of hypoxia-related circRNAs was analyzed using RNA sequencing. A unique circRNA termed circRNF13 was found to be upregulated in PC tissues and may be a potential prognostic indicator. HIF-1α and EIF4A3 are involved in regulating the biogenesis of circRNF13. Furthermore, circRNF13 was validated to exert a stimulative effect on cell proliferation, angiogenesis, invasion and glycolysis. Importantly, we found that circRNF13 promoted PDK3 levels by acting as a miR-654-3p sponge, thus promoting the PC malignant process. Collectively, our results reveal that hypoxia-induced circRNF13 mediated by HIF-1α and EIF4A3 promotes tumor progression and glycolysis in PC, indicating the potential of circRNF13 as a prognostic biomarker and therapeutic target for PC.

## Cancer: circular RNA in pancreatic tumor progression

The low-oxygen (hypoxic) conditions within pancreatic tumors promote production of a newly identified circular RNA molecule associated with aggressive tumor growth. Pancreatic cancers are generally difficult to treat, and the hypoxic conditions of the tumor interior are known to contribute to greater resistance to chemotherapy. Several studies have indicated that these conditions also lead to greater production of certain circular RNAs, and researchers led by Rong Wan at Shanghai Jiao Tong University School of Medicine, China, set out to identify specific RNA molecules produced within hypoxic pancreatic tumor tissue. Biopsy samples containing high levels of one such RNA, circRNF13, were consistently linked with a poor prognosis. The researchers also identified downstream targets modulated by circRNF13 that contribute to progression, which could offer new targets for drug development.

## Introduction

Pancreatic cancer (PC) continues to have a poor prognosis and is the seventh leading cause of cancer death^1^. Due to the lack of early symptoms, most patients are diagnosed in the advanced stages of the disease, and the 5-year overall survival rate is only 9%^2^. The incidence and mortality rates of pancreatic cancer are almost identical, and it has been predicted to be the third most important cause of cancer-related death by 2025 in European countries^3^. Thus, identifying the mechanisms of PC tumorigenesis and developing effective therapeutic targets are urgently needed.

Apart from a high frequency of *KRAS* gene mutations, PC demonstrates extensive stromal tissue and a severe intratumoral hypoxic microenvironment^4,5^. The presence of desmoplasia and hypoxia contributes to resistance to chemotherapy and is associated with tumor progression and poor prognosis^6,7^. In hypoxia, PC cells exhibit more aerobic glycolysis rather than mitochondrial oxidative phosphorylation (OXPHOS) to supply metabolic and bioenergetic materials, which is known as the Warburg effect^8,9^. The central player in aerobic glycolysis is hypoxia-inducible factor-1 (HIF-1), which determines whether glucose is consumed via OXPHOS or glycolysis^10,11^. During normoxia, HIF-1α interacts with von Hippel–Lindau (VHL) protein and is subject to proteasomal degradation, whereas HIF-1α dimerizes with HIF-β and accumulates in the nucleus under hypoxic conditions. Then, HIF-1α transactivates hundreds of genes that enhance aerobic glycolysis and the progression of tumor cells^12,13^.

Circular RNAs (circRNAs) are a kind of covalently closed noncoding RNA formed by RNA back splicing. They are mainly composed of exons and/or introns and widely exist in eukaryotic cells^14^. CircRNAs have been proven to be involved in multiple cancer biological processes, including cell proliferation, migration and angiogenesis^15,16^. However, the expressional regulation of circRNAs has not been well explored. A recent report indicated that numerous circRNAs were regulated by the splicing factor Quaking (QKI) under exposure to TGF-β^17^. Since a series of protein-coding genes can be transactivated by HIF-1α under hypoxia, whether circRNAs are regulated in response to hypoxia is far from clear. Jes-Niels et al. identified abundant circRNAs in endothelial cells cultured under hypoxic conditions. Their study demonstrated that hypoxia-induced cZNF292 could regulate angiogenic sprouting and cell proliferation^18^. Furthermore, Yang et al. implicated that circ-133 was hypoxia inducible, which was shown to promote tumor progression^19^. However, there are still many other unknown circRNAs that may be involved in molecular pathways that respond to hypoxia, and their function in regulating cellular adaptation remains to be further explored.

In the present study, high-throughput RNA sequencing (RNA-seq) was performed on hypoxic and normoxic cultured PC cells. A novel circRNA, circRNF13 (hsa_circ_0001346), was found to be upregulated in PC tissues and was a potential prognostic indicator. Moreover, HIF-1α and EIF4A3 may be involved in regulating the biogenesis of circRNF13. Then, circRNF13 was validated to exert a stimulative effect on cell proliferation, angiogenesis, invasion and glycolysis. Importantly, we found that circRNF13 promoted PDK3 levels by acting as a miR-654-3p sponge, thus promoting the PC malignant process. Our study describes a novel HIF-1α/circRNF13/miR-654-3p/PDK3 regulation mechanism and provides potential prognostic and diagnostic indicators.

## Materials and methods

### Cell lines and tissue microarrays (TMAs)

Human pancreatic cancer cell lines (MIA PaCa-2, SW-1990, PANC-1, AsPC-1, BxPC-3) and normal pancreatic ductal epithelial cells (HPDE6-c7) were purchased from the Type Culture Collection of the Chinese Academy of Science (Shanghai, China). MIA PaCa-2, PANC-1, BxPC-3 and HPDE6-c7 cells were cultured in DMEM supplemented with 10% fetal bovine serum (FBS). SW-1990 and AsPC-1 cells were cultured in RPMI 1640 medium with 10% FBS. TMAs containing 90 pairs of pancreatic cancer samples were purchased from Shanghai Outdo Biotech (Shanghai, China), and the application of TMAs was approved by the Ethics Committee of Shanghai General Hospital.

### Real-time PCR (RT–PCR) and Western blotting analysis

RT–PCR and Western blotting methods were described previously^20^. The primary antibodies used in our study were as follows: β-Actin (CST, #4970), GLUT1 (Abcam, ab115730), HK-2 (CST, #2867), PKM2 (CST, #4053), LDHA (CST, #3582), PDK1 (CST, #3062) and PDK3 (Abcam, ab154549).

### Establishment of stable overexpression and knockdown cell lines

SW-1990 cells with stable circRNF13 overexpression were generated by using the lentivirus vector pGLV31H1-Luci. Sh-circRNF13 and sh-NC lentiviruses were purchased from OBiO Biotechnology (Shanghai, China) and cloned into MIA PaCa-2 cell lines. The transfected cells were selected with puromycin (Sigma, USA) for 2 weeks to generate stable cell lines.

### Colony formation assay

A total of 800 cells were seeded in 6-well plates. After 9–14 days, the proliferating colonies were washed three times with PBS, fixed with 4% paraformaldehyde for 30 min and then stained with 0.1% crystal violet. The number of colonies was counted by ImageJ software.

### 5-Ethynyl-2'-deoxyuridine (EdU) assay

Cells were cultured in 24-well plates and then incubated in EdU reagent (RiboBio, Gauangzhou, China) for 2 h. After that, the cells were fixed, permeabilized and EdU stained. Following nuclear staining with Hoechst 33342, EdU-positive cells were analyzed under a fluorescence microscope.

### Angiogenesis assay

The cells were inoculated into 6-well plates. After being attached to the wall, the cells were washed twice with PBS and cultured in serum-free medium for 24 h, and the supernatant was collected. The matrix glue was poured onto the precooled 96-well plate in advance. Human umbilical vein endothelial cells (HUVECs) were resuspended with the supernatant collected before, and 3 × 10^4^ cells were added to the 96-well plate and incubated for 6 h. Then, five fields were randomly selected under a microscope to observe and count the number of tubes.

### Fluorescence in situ hybridization (FISH)

To determine the location of circRNF13 and miR-654-3p, hsa-circ-0001346-digoxin-FITC (5'-GTTGTAAAATCACCTTTCTTGAATTTATGTA-3') and hsa-miR-654-3p-biotin-CY3 probes (5'-AAGGTGATGGTCAGCAGACATA-3') were designed and synthesized by Geneseed (Guangzhou, China). Cells attached to slides were fixed with 4% paraformaldehyde for 10 min, washed with PBS, and incubated in 0.5% Triton X-100 for 15 min. Then, hybridization was carried out with circ-0001346 and miR-654-3p FISH probes overnight at 37 °C. The next day, after blocking with 3% BSA for 30 min, diluted digoxin-FITC and biotin-Cy3 fluorescent secondary antibodies were added to the slides and incubated for 1 h. Cell nuclei were stained with DAPI-antifade solution. Images were captured using a confocal microscope (Leica, Germany).

### In situ hybridization (ISH)

An ISH assay was used to detect the level of circ-0001346 in TMA. The method was described previously^20^.

### Luciferase reporter assay

For analysis of RNF13 promoter activity, wild-type and mutant RNF13 promoter reporter plasmids were synthesized by RiboBio (Guangzhou, China). The cells were cotransfected with empty vector or HIF-1α with promoter luciferase reporters. Other luciferase reporter plasmids (pmirGLO-hsa-circ-0001346-WT, pmirGLO-hsa-circ-0001346-MUT, PmirGLO-PDK3-3'UTR-WT and PmirGLO-PDK3-3'UTR-MUT) were also synthesized by RiboBio. Cells were cotransfected with wild-type or mutated circ-0001346 or PDK3 3'-UTR reporter plasmids and with miR-654-3p mimics or negative controls using Lipofectamine™2000 (Invitrogen). After 48 h of transfection, firefly and Renilla luciferase activities were determined using a Dual Luciferase Assay Kit (Promega, USA).

### Extracellular acidification rate (ECAR) and oxygen consumption rate (OCR) assays

ECAR and OCR were measured using the Seahorse XF96 extracellular flux analyzer (Seahorse Bioscience). A total of 1 × 10^4^ cells were seeded in an XF96 plate and incubated overnight. ECAR was measured using a Seahorse XF Glycolysis Stress Test kit (Agilent Technologies), and OCR was detected by a Seahorse XF Cell Mito Stress Test kit (Agilent Technologies) according to the method described previously^21^.

### Biotinylated RNA pull-down assay

To verify whether EIF4A3 could bind to the flanking regions of circRNF13, biotinylated circRNF13 flanking RNA sequence probes were synthesized. The probes were incubated with streptavidin magnetic beads to generate probe-coated beads. Then, the probe-coated beads were incubated with MIA PaCa-2 cell lysates overnight. After elution and purification, the proteins were validated by Western blotting. For circRNF13 pulled down target miRNAs, the biotinylated circRNF13 probe was incubated with streptavidin beads and MIA PaCa-2 and SW-1990 cell lysates, eluted with wash buffer and analyzed by qRT–PCR.

### Chromatin immunoprecipitation (ChIP)

ChIP experiments were conducted using a SimpleChIP Enzymatic Chromatin IP Kit (CST, USA) according to the manufacturer’s instructions. Briefly, 1 × 10^7^ PC cells were cross-linked with 1% formaldehyde. Then, the nuclear precipitate was fragmented using enzymatic digestion combined with sonication. After centrifugation, the supernatants were collected and incubated with antibodies overnight. Then, the chromatin antibody complexes were de-crosslinked and enriched DNA was purified, followed by qRT–PCR analysis.

### RNA immunoprecipitation (RIP)

RIP assays were performed using an RNA Immunoprecipitation Kit (Geneseed). A total of 1 × 10^7^ cells were lysed in RIP lysis buffer and incubated with protein A + G beads. Then, the cell lysates were incubated with antibodies against EIF4A3 and IgG containing protein A + G beads. The final purified RNA was subjected to qRT–PCR analysis.

### Immunohistochemistry (IHC)

Before embedding in paraffin, tissue samples were fixed with 4% paraformaldehyde and sectioned. Then, the sections were incubated with primary antibodies at 4 °C overnight, followed by incubation with secondary antibody. For histological analysis, the sections were stained with DAB and hematoxylin. The IHC score was obtained by multiplying the staining intensity (0 = negative, 1 = weak-positive, 2 = moderate-positive, 3 = strong-positive) by the staining area (0 = 0–9%, 1 = 10–24%, 2 = 25–49%, 3 = 50–74%, 4 = 75–100%). The score was assessed by three proficient pathologists independently. An IHC score ≤ 4 indicated low expression, while >4 defined high expression.

### Animal models

For the in vivo tumor growth study, four-week-old male BALB/c nude mice were randomly divided into four groups (*n* = 5). The cells (1.0 × 10^7^) were subcutaneously injected into the armpits of nude mice. Volumes of tumor ((length × width^2^)/2) were measured twice a week. Subcutaneous tumors were removed and weighed 21 days later.

For the in vivo lung metastasis model, the nude mice were also divided into four groups (*n* = 5). Cells were injected via the tail vein. Metastasis of tumor cells in vivo was observed every 2 weeks using an IVIS Illumina System (Caliper Life Sciences, USA). After 6 weeks, the lungs were removed and stained with hematoxylin and eosin (HE) to observe the metastatic nodules. Animal assays in this study were approved by the Animal Ethics Committee of Shanghai General Hospital (IACUC approval No. 2019AW048).

For the hepatic metastasis model, a longitudinal incision was made in the left abdomen of nude mice after the mice were anesthetized. A total of 1.0 × 10^6^ cells were injected into the spleen. Then, the incision was sutured. Six weeks later, the mice were sacrificed, and the livers were removed and photographed. Subsequently, the livers were fixed with 4% paraformaldehyde and stained with HE.

### Statistical analysis

Data from at least three independent experiments are presented as the means ± SDs and were statistically analyzed using SPSS 20.0. Chi-square tests (*χ*^2^ tests) were used to analyze the relationships between nonparametric variables. Student’s *t*-test or one-way ANOVA was performed in GraphPad Prisma 7 to evaluate the relationship between parametric variables. Survival differences were evaluated using the Kaplan–Meier method. *P* < 0.05 was considered statistically significant.

## Results

### CircRNA expression profiles in hypoxia-induced PC cells

**Fig. 1:**
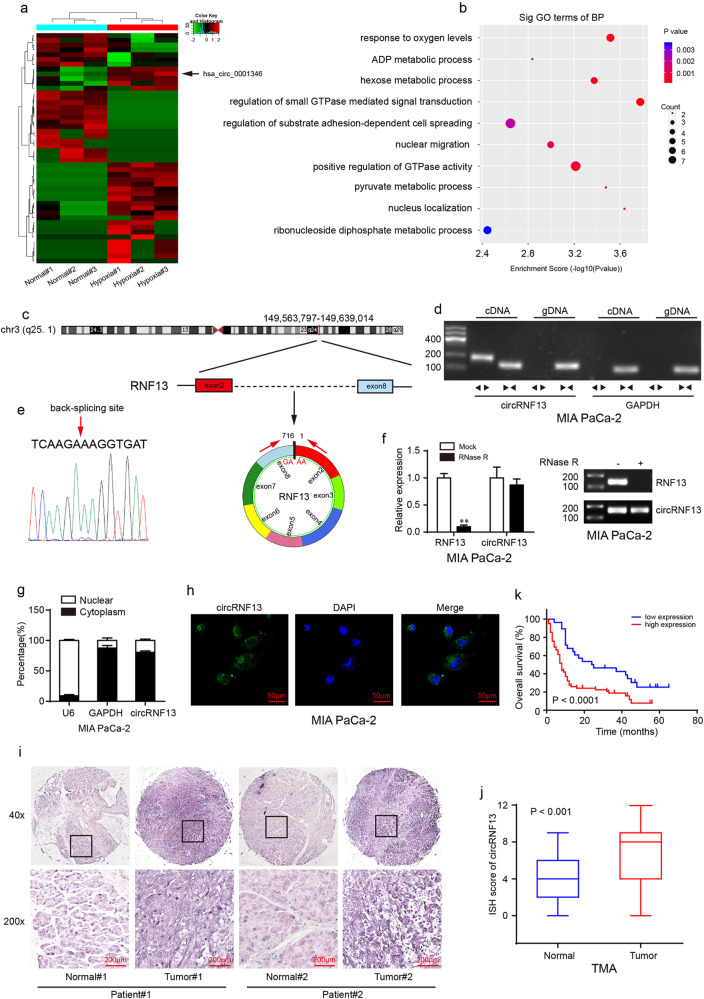
Expression profiles of circRNAs in hypoxia-induced PC cells. **a** Hierarchical clustering of 48 significantly differentially expressed circRNAs in MIA PaCa-2 cells under normal and hypoxic conditions. **b** GO analysis showing that these differentially expressed circRNAs and mRNAs were enriched in the energy metabolic process. **c** Schematic illustration of the genomic loci of the circRNF13 gene. **d** circRNF13 was amplified by divergent primers in cDNA rather than in gDNA. GAPDH was used as a linear control. **e** Sanger sequencing confirmed the back-spliced junction of circRNF13. **f** qRT–PCR detection of the relative expression of circRNF13 and RNF13 in the presence or absence of RNase R. **g** Nuclear-cytoplasmic fractionation assay showing the location of circRNF13. **h** FISH assay confirmed that circRNF13 was mainly located in the cytoplasm (magnification: 400x, scale bar: 50 μm). **i** Representative ISH images of circRNF13 expression in PC tissues and adjacent normal tissues (scale bar: 200 μm, magnification: top 40x, bottom 200x). **j** ISH scores of circRNF13 in 90 PC tissues and corresponding normal tissues. **k** Kaplan–Meier survival analysis was used to evaluate the 5-year OS in the circRNF13 low group and circRNF13 high group. Data represent at least three independent experiments and are presented as the means ± SD. *P < 0.05, **P < 0.01.

To explore crucial circRNAs in hypoxia-induced pancreatic cancer cells, high RNA-seq was performed in hypoxia-cultured and normal cultured pancreatic cancer cells (MIA PaCa-2). After screening, there were 26 upregulated and 22 downregulated circRNAs (fold change > 2.0 and *P* < 0.05) in total (Fig. 1a). GO (Gene Ontology) analysis for these upregulated circRNAs/mRNAs demonstrated that they were mainly associated with the energy metabolic process (Fig. 1b). To further verify the RNA-Seq results, qRT–PCR was performed to measure the expression of the top ten upregulated circRNAs in hypoxia-cultured MIA PaCa-2 cells compared with normal cultured cells. In agreement with the RNA-Seq results, hsa_circ_0006719, hsa_circ_0002483, hsa_circ_0007843, hsa_circ_0105820, hsa_circ_0001346, and hsa_circ_0117799 were significantly increased under hypoxia (Supplementary Fig. 1a). As hsa_circ_0001346 was the most upregulated circRNA, we selected it for further study. Hsa_circ_0001346, spliced from the RNF13 gene, was located at chr3:149,563,797–149,639,014 and formed by the head-to-tail splicing of exon 2 and exon 8 (Fig. 1c). To detect the circular characteristics of circRNF13, convergent primers and divergent primers were designed. PCR results showed that divergent primers could only amplify products from cDNA but not from gDNA in MIA PaCa-2 cells (Fig. 1d). Sanger sequencing confirmed the back-spliced junction of circRNF13 (Fig. 1e). RNase R is a highly processive 3′ to 5′ exoribonuclease that can digest linear RNAs^22^. Our results showed that circRNF13 could resist RNase R digestion, while the linear form of RNF13 was dramatically decreased (Fig. 1f), indicating that circRNF13 consisted of a loop structure. Subsequently, nuclear-cytoplasmic fractionation and FISH assays showed that circRNF13 was mainly detected in the cytoplasm (Fig. 1g, h). These results demonstrate that circRNF13 is a circular RNA that is localized predominantly in the cytoplasm.

### CircRNF13 is overexpressed in PC

Next, we determined the expression of circRNF13 in PC cells, and the results showed that circRNF13 expression was higher in PC cells than in normal pancreatic ductal epithelial cells (HPDE6-c7) (Supplementary Fig. 1b). Based on this result, we selected MIA PaCa-2 cells, which showed the highest expression of circRNF13, and SW-1990 cells, which showed the lowest expression of circRNF13. To further determine the expression of circRNF13, ISH analysis of the TMA containing 90 pancreatic cancer tissues and adjacent normal tissues was conducted. The results indicated that circRNF13 expression was significantly higher in PC tissues than in corresponding normal tissues (Fig. 1i, j). Subsequently, we analyzed the relationship between circRNF13 levels and clinicopathological features. It was found that circRNF13 expression was higher in PC tissues of T3-T4 stage, N1 stage, M1 stage and AJCC stage IIB-IV than in those of T1-T2 stage, N0 stage, M0 stage and AJCC-IIA stage and was positively correlated with T stage (*P* = 0.001), N stage (*P* < 0.0001), M stage (*P* = 0.01) and AJCC stage (*P* < 0.0001) but not with sex, age or pathologic grade (Supplementary Table 1 and Supplementary Fig. 1c). Kaplan–Meier survival analysis revealed that patients with higher circRNF13 expression had worse overall survival (OS) (Fig. 1k). Taken together, circRNF13 could serve as a potential diagnostic and prognostic factor for PC patients.

### CircRNF13 is hypoxia-inducible and regulated by HIF-1α and EIF4A3

**Fig. 2:**
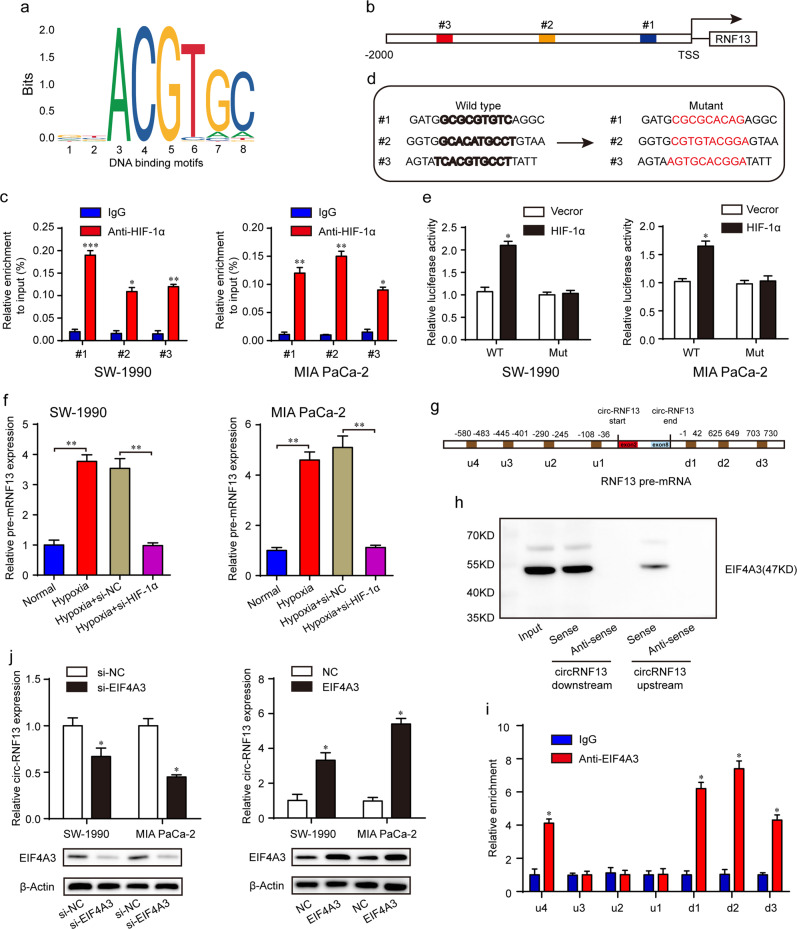
HIF-1α and EIF4A3 regulate circRNF13 expression. **a** DNA-binding motifs of HIF-1α on the promoters of RNF13. **b** Schematic diagram of the putative binding sites of HIF-1α on the RNF13 promoter. **c** ChIP–qPCR experiments were conducted to determine the binding of HIF-1α to the RNF13 promoter using an anti-HIF-1α antibody. **d** Schematic illustration of wild-type and mutant reporter plasmids. **e** Relative luciferase activities in SW-1990 and MIA PaCa-2 cells transfected with luciferase reporter plasmids containing the wild-type or mutant RNF13 promoter sequence and HIF-1α overexpression plasmids. **f** qRT–PCR analysis of the relative expression of pre-mRNA and mRNA of RNF13 under hypoxia. **g** The CircInteractome database was used to predict the binding sites of EIF4A3 in the downstream and upstream regions of circRNF13 pre-mRNA. **h** RNA pull-down assay determined that EIF4A3 could bind to both downstream and upstream sequences. **i** RIP-qPCR confirmed the binding sites of EIF4A3 in the flanking sequence of circRNF13. **j** qRT–PCR analysis of circRNF13 expression after EIF4A3 overexpression and knockdown in PC cells. Data represent at least three independent experiments and are presented as the means ± SD. *P < 0.05, **P < 0.01, ***P  < 0.001.

Consistent with the results in MIA PaCa-2 cells, circRNF13 was also overexpressed in SW-1990 cells in response to hypoxia (Supplementary Fig. 2a). However, the circRNF13 level was completely reversed after HIF-1α knockdown (Supplementary Fig. 2b). To detect how HIF-1α regulates circRNF13 in PC, we analyzed the genomic signatures of RNF13 and found 3 putative HIF-1α-binding sites on the RNF13 promoter through the JASPAR database (Fig. 2a, b). The ChIP–qPCR results indicated that HIF-1α could interact with the RNF13 promoter (Fig. 2c). Furthermore, wild type (WT) reporter plasmids containing the full-length RNF13 promoter and mutant (Mut) reporter plasmids were constructed (Fig. 2d). A luciferase reporter assay demonstrated that HIF-1α could increase the luciferase activity of WT plasmids but not reporter plasmids (Fig. 2e). Then, we detected the pre-mRNA and mRNA levels of RNF13 under hypoxia. Hypoxia stimulation promoted pre-mRNA levels of RNF13 in PC cells, and HIF-1α knockdown decreased RNF13 pre-mRNA expression (Fig. 2f). However, there were no significant changes in RNF13 mRNA and protein levels under hypoxia (data not shown). We speculated that HIF-1α promotes RNF13 transcription by binding to the RNF13 promoter, thus promoting RNF13 pre-mRNA generation. Recent studies have shown that circRNAs are regulated by some RNA-binding proteins, such as muscleblind, QKI, hnRNPs, and FUS^17,23–25^. Several studies also discovered that EIF4A3, a major component of the exon junction complex, could bind to flanking intron sequences of pre-mRNA, inducing the formation of circRNAs^26,27^. We found 7 putative binding sites of EIF4A3 matching the flanking regions of circRNF13 through CircInteractome (Fig. 2g). Then, probes for biotin-labeled downstream and upstream flanking sequences of circRNF13 were designed. RNA pull-down assays showed that EIF4A3 could bind to both downstream and upstream sequences (Fig. 2h). A RIP-qPCR assay was further conducted to detect whether EIF4A3 could bind to these predicted sites using EIF4A3 antibody. The results demonstrated that EIF4A3 was enriched in 3 downstream binding fragments and 1 upstream binding site (Fig. 2i). In addition, EIF4A3 overexpression induced circRNF13 expression, while EIF4A3 knockdown led to a reduction in circRNF13 expression (Fig. 2j). These data revealed that EIF4A3 interacted with the RNF13 pre-mRNA regions, controlling the biogenesis of circRNF13.

### CircRNF13 promotes PC cell proliferation and angiogenesis

**Fig. 3:**
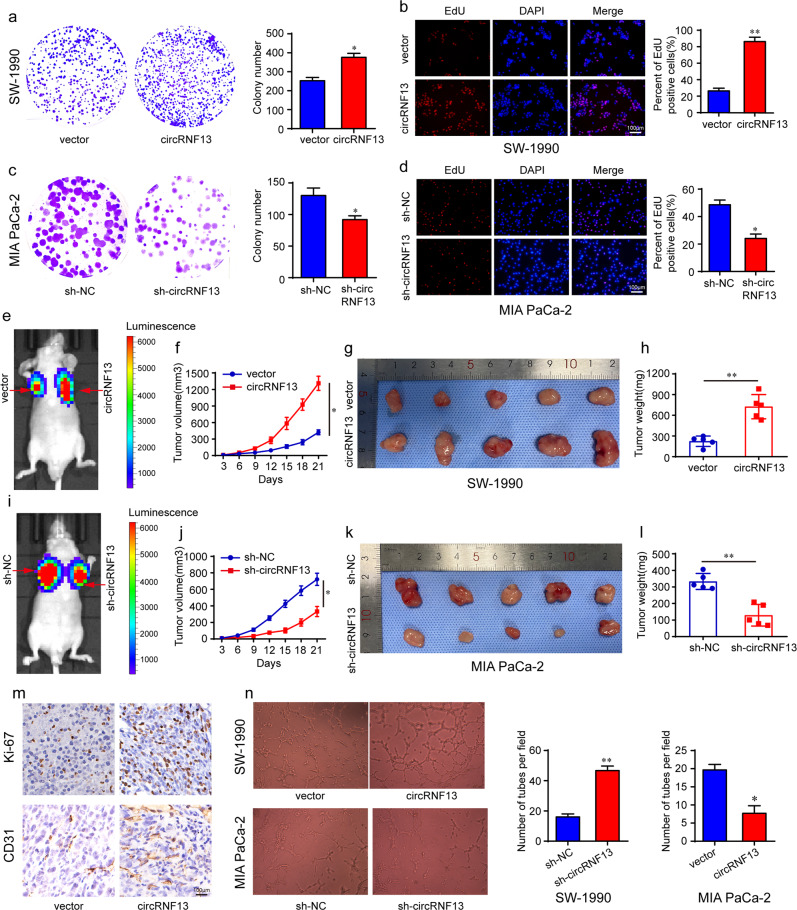
CircRNF13 promotes PC proliferation and angiogenesis in vitro and in vivo. **a**, **c** A colony formation assay was conducted to determine the proliferation ability of SW-1990 and MIA PaCa-2 cells after circRNF13 was overexpressed or knocked down. **b**, **d** EdU assay further confirmed the proliferation ability of SW-1990 and MIA PaCa-2 cells after circRNF13 was overexpressed or knocked down (scale bar: 100 μm, magnification: 200x). **e**, **i** Representative images of the tumor xenograft model of each group were obtained by bioluminescence imaging. **f**, **j** The line graphs show the growth of tumors as monitored every 3 days. **g**, **k** The subcutaneous tumors were removed after 21 days. **h**, **l** The weights of tumors were evaluated. **m** Ki-67 and CD31 expression in tumor tissues of the circRNF13-overexpressing group and control group (scale bar: 100 μm, magnification: 200x). **n** A tube formation assay was conducted to confirm the effect of circRNF13 on angiogenesis. Data represent at least three independent experiments and are presented as the means ± SD. *P < 0.05, **P < 0.01.

To elucidate the function of circRNF13 on cell biological behavior, stably circRNF13-overexpressing SW-1990 cells were established (Supplementary Fig. 3a). CircRNF13 upregulation clearly promoted PC cell colony formation efficiency (Fig. 3a) and the percentage of EdU-positive cells (Fig. 3b). We also generated stable circRNF13-knockdown MIA PaCa-2 cells (Supplementary Fig. 3b). As expected, knockdown of circRNF13 significantly suppressed PC cell proliferation (Fig. 3c, d). In addition, we established a tumor xenograft model to verify the role of circRNF13 in vivo. The results showed that circRNF13 overexpression significantly accelerated tumor growth with prominently higher tumor volume and final tumor weight (Fig. 3e–h), while the volume and weight of tumors in the circRNF13 knockdown group were lower than those in the control group (Fig. 3i–l). After removing the subcutaneous tumor tissues, IHC analysis was performed. The results revealed that Ki-67 expression was higher in circRNF13-overexpressing tumors than in control tumors (Fig. 3m). Tumor growth is often accompanied by neovascularization^28^. Then, we detected the expression of CD31, which is a biomarker of angiogenesis. The results demonstrated that the percentage of CD31-positive cells was higher in circRNF13-overexpressing tumors than in control tumors (Fig. 3m). Conversely, the Ki-67 and CD31 levels were dramatically decreased in circRNF13 knockdown tumors (Supplementary Fig. 3b). To further determine the function of circRNF13 in PC angiogenesis, a tube formation assay was conducted. The results showed that tube formation of human umbilical vein endothelial cells (HUVECs) was increased by conditioned medium from circRNF13-overexpressing SW-1990 cells versus conditioned medium from negative control cells (Fig. 3n). Correspondingly, angiogenic capabilities were largely impaired by conditioned medium from circRNF13 knockdown MIA PaCa-2 cells (Fig. 3n). In summary, these results suggest that circRNF13 plays an important role in PC proliferation and angiogenesis.

### CircRNF13 promotes PC metastasis in vitro and in vivo

**Fig. 4:**
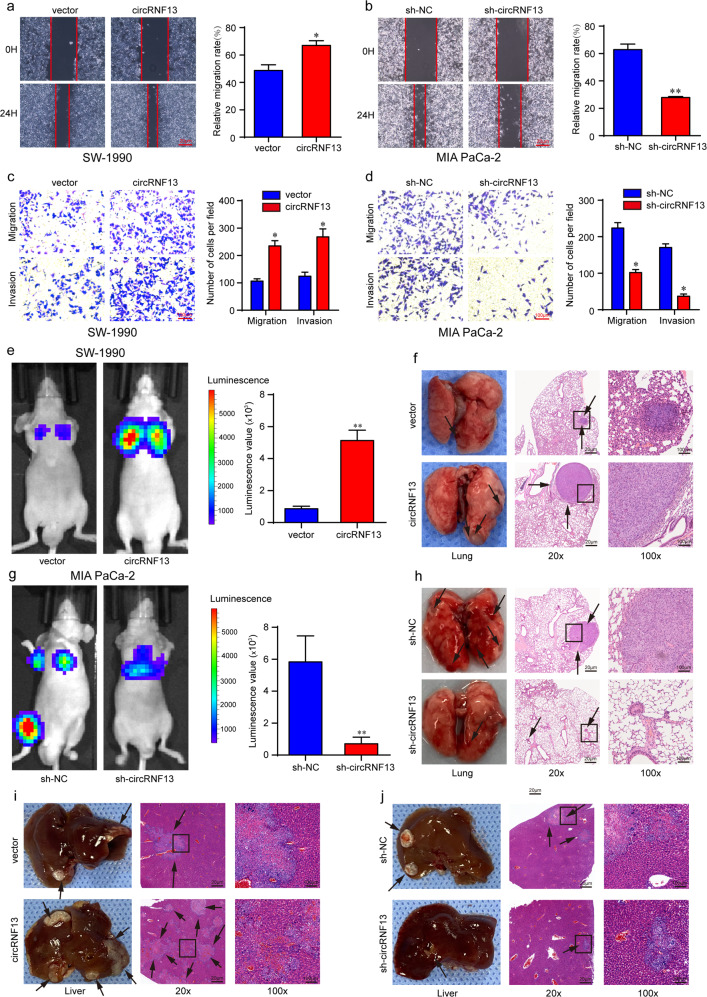
CircRNF13 promotes PC metastasis in vitro and in vivo. **a**–**d** Cell migration and invasion abilities were assessed by wound healing (scale bar: 20 μm) and transwell assays (scale bar: 100 μm) in SW-1990 and MIA PaCa-2 cells after circRNF13 was overexpressed or knocked down. **e**, **g** Representative bioluminescence images of mice 8 weeks after tail vein injection of SW-1990/circRNF13 and MIA PaCa-2/sh-circRNF13 cells. **f**, **h** Representative images of metastatic nodes in the lungs and HE staining of the lung tissues of the respective groups. **i**, **j** Representative liver images and HE staining of the liver tissues of the respective groups. Data represent at least three independent experiments and are presented as the means ± SD. *P < 0.05, **P < 0.01.

Next, the roles of circRNF13 in PC metastasis were explored. The wound-healing assay revealed that forced expression of circRNF13 increased the mobility of SW-1990 cells and that circRNF13 depletion in MIA PaCa-2 cells produced the opposite effects (Fig. 4a, b). Transwell migration and Matrigel invasion assays further demonstrated that circRNF13 overexpression promoted the migration and invasion ability of SW-1990 cells (Fig. 4c, d). Correspondingly, circRNF13 knockdown had the opposite effects (Fig. 4c, d). Next, a lung metastasis model was established to assess the function of circRNF13 in PC metastasis. As shown in Fig. 4e, g and Supplementary Fig. 4a, c, upregulation of circRNF13 increased lung metastasis, while circRNF13 knockdown decreased lung metastasis compared with their respective controls. Consistent with the bioluminescence imaging, the number of metastatic nodules in circRNF13-overexpressing mice was much higher than that in control mice (Fig. 4f and Supplementary Fig. 4b). In contrast, lungs with circRNF13 knockdown formed fewer metastatic nodules than control lungs (Fig. 4h and Supplementary Fig. 4d). In addition, the liver metastasis model was also established by splenic injection. Consistent with the lung metastasis results, more liver metastases were observed in mice injected with circRNF13-overexpressing cells (Fig. 4i and Supplementary Fig. 4e), while circRNF13 depletion significantly attenuated liver metastasis (Fig. 4j and Supplementary Fig. 4f). Collectively, our results demonstrate that circRNF13 may act as an oncogene to facilitate the metastasis of PC.

### CircRNF13 silencing suppresses the tumor-promoting effects of hypoxia in PC cells

**Fig. 5:**
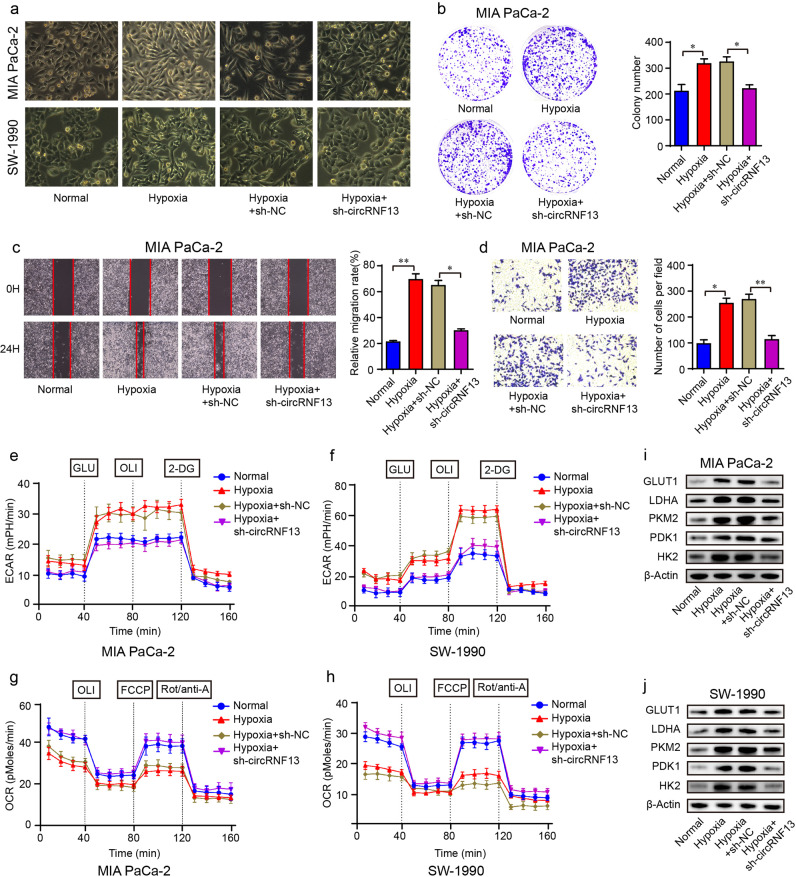
Inhibition of circRNF13 suppresses the tumor-promoting effects of hypoxia in PC cells. **a** Morphological changes in SW-1990 and MIA PaCa-2 cells. **b** The effect of circRNF13 silencing on hypoxia-induced cell proliferation was detected by a colony formation assay. **c**, **d** Wound healing and transwell assays were conducted to detect the migration and invasion abilities. **e**, **f** ECARs in SW-1990 and MIA PaCa-2 cells were measured by the Seahorse XF96 extracellular Flux analyzer. **g**, **h** OCRs in SW-1990 and MIA PaCa-2 cells were measured by a Seahorse XF96 extracellular flux analyzer. **i**, **j** Western blotting was conducted to analyze the changes in several glucose metabolism-related enzymes in SW-1990 and MIA PaCa-2 cells. Data represent at least three independent experiments and are presented as the means ± SD. *P < 0.05, **P < 0.01.

Hypoxia is a well-recognized hallmark of solid tumors and is involved in cancer progression^29^. In our study, we found that oval-shaped PC cells elongated and became spindle-like mesenchymal-shaped cells under hypoxia. In contrast, no manifest changes were observed in PC cell morphology under hypoxia along with circRNF13 knockdown (Fig. 5a). In addition to the observed morphological changes, circRNF13 silencing significantly delayed the SW-1990 and MIA PaCa-2 cell colony formation efficiency caused by hypoxia (Fig. 5b and Supplementary Fig. 5a). Moreover, hypoxia failed to enhance the migration and invasiveness abilities of PC cells when circRNF13 was knocked down (Fig. 5c, d and Supplementary Fig. 5b, c). Collectively, these findings reveal that circRNF13 is critical for the hypoxia responses of PC cells in proliferation, migration and invasion.

Glycolysis is the primary metabolic pathway in hypoxia, characterized by excessive glucose uptake and lactate production. To assess the influence of circRNF13 on glycolysis and oxidative phosphorylation, the extracellular acidification rate (ECAR) and oxygen consumption rate (OCR) were measured. The Seahorse XF96 stress test demonstrated that hypoxia increased glycolysis and glycolytic capacity in MIA PaCa-2 and SW-1990 cells, whereas these capacities were reduced by circRNF13 silencing (Fig. 5e, f and Supplementary Fig. 5d, e). Moreover, oxidative phosphorylation significantly decreased under hypoxia, as reflected by basal oxygen consumption and respiration capacity, while circRNF13 knockdown reversed the OCR at both basal and maximal levels (Fig. 5g, h and Supplementary Fig. 5f, g). To further explore the mechanism by which circRNF13 regulates glycolysis, several glucose metabolism-related enzymes were examined. Western blotting results demonstrated that hypoxia obviously increased the protein levels of glucose transporter-1 (GLUT1), lactate dehydrogenase A (LDHA), pyruvate kinase M 2 (PKM2), pyruvate dehydrogenase kinase 1 (PDK1) and hexokinase II (HK2). However, these proteins in circRNF13 knockdown cells did not exhibit significant changes under hypoxic stimulation (Fig. 5i, j). Therefore, we concluded that circRNF13 was crucial in glycolytic metabolism under hypoxia.

### CircRNF13 acts as a miRNA sponge for miR-654-3p in PC cells

**Fig. 6:**
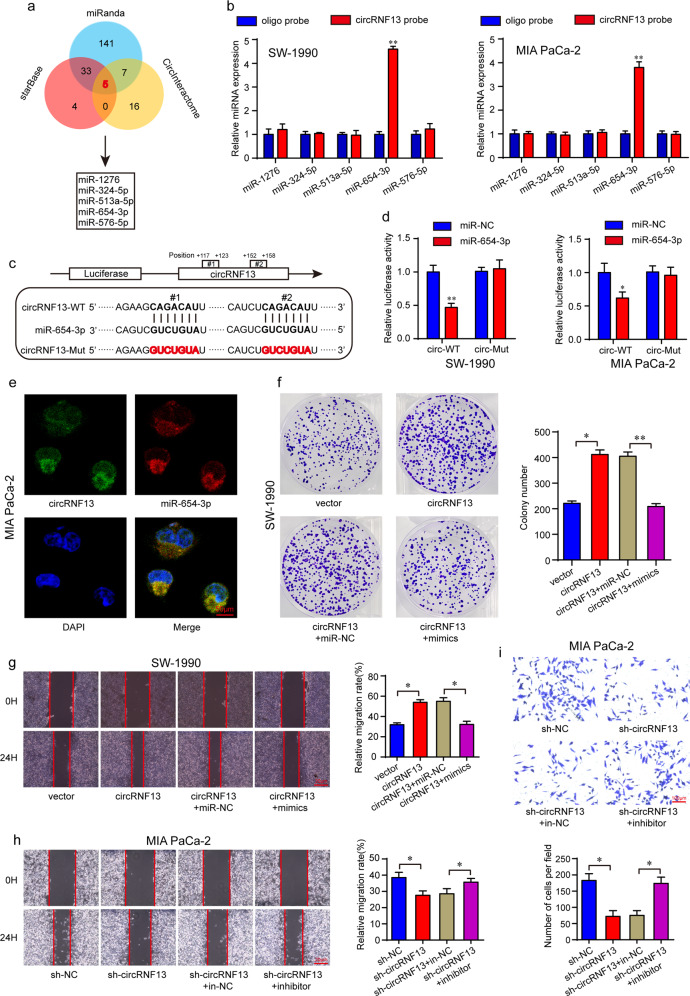
CircRNF13 serves as a sponge for miR-654-3p in PC cells. **a** Bioinformatics analysis of the target miRNAs of circRNF13. **b** An RNA pulldown assay was performed to detect the enrichment of circRNF13 and its potential target miRNAs. **c** Schematic illustration of circRNF13 wild-type (WT) and mutant (Mut) luciferase reporter plasmids. **d** The luciferase reporter assay was performed in SW-1990 and MIA PaCa-2 cells cotransfected with circRNF13 and miR-654-3p mimics. **e** FISH showing that circRNF13 colocalized with miR-654-3p in PC cells (magnification: 400x, scale bar: 50 μm). **f**, **g** Cell proliferation and migration abilities were detected in circRNF13-overexpressing SW-1990 cells transfected with miR-654-3p mimics or mimics NC (miR-NC) by colony formation and wound healing assays, respectively. **h**, **i** Cell migration and invasion abilities were determined in circRNF13 knockdown MIA PaCa-2 cells transfected with miR-654-3p inhibitor or inhibitor NC (in-NC). Data represent at least three independent experiments and are presented as the means ± SD. *P < 0.05, **P < 0.01.

CircRNAs have been reported to function mainly as miRNA sponges and subsequently regulate gene expression^30^. Given that circRNF13 is stable in the cytoplasm, we examined whether circRNF13 could bind to miRNAs. Bioinformatics analysis using starBase, miRanda and CircInteractome predicted 5 potential miRNAs that may bind to circRNF13: miR-1276, miR-324-5p, miR-513a-5p, miR-576-5p and miR-654-3p (Fig. 6a). To investigate these potential target miRNAs, a 3′‐terminal-biotinylated circRNF13 probe was designed. We found that only miR-654-3p was abundantly pulled down by the circRNF13 probe in PC cells, whereas other miRNAs had no enrichment (Fig. 6b). In addition, miR-654-3p has been implicated in varying types of cancers and acts as a tumor suppressor^31–34^. To further validate this prediction, luciferase reporter plasmids were constructed (Fig. 6c). The luciferase reporter assay results indicated that miR-654-3p overexpression was able to suppress the luciferase activity of the wild-type luciferase reporter plasmid containing the complete circRNF13 sequence, but transfection with miR-654-3p mimics had no significant effect on the luciferase activity of the mutant plasmid in which the corresponding binding sites were mutated (Fig. 6d). It has been proven that the miRNA sponge process occurs in the cytoplasm. Then, a FISH assay was performed in MIA PaCa-2 cells to observe the subcellular localization of circRNF13 and miR-654-3p. As shown in (Fig. 6e), circRNF13 and miR-654-3p were mostly colocalized in the cytoplasm. To further confirm the function of circRNF13 on miR-654-3p, rescue experiments were conducted using miR-654-3p mimics and inhibitor. The results showed that miR-654-3p mimics significantly decreased the promoting effect of circRNF13 on cell proliferation, migration and invasion (Fig. 6f, g and Supplementary Fig. 6a). Furthermore, the miR-654-3p inhibitor rescued the colony forming ability, migration and invasion abilities of circRNF13 knockdown PC cells (Fig. 6h, i and Supplementary Fig. 6b). These results demonstrated that circRNF13 could function as a sponge for miR-654-3p.

### CircRNF13 modulates the miR-654-3p target PDK3

**Fig. 7:**
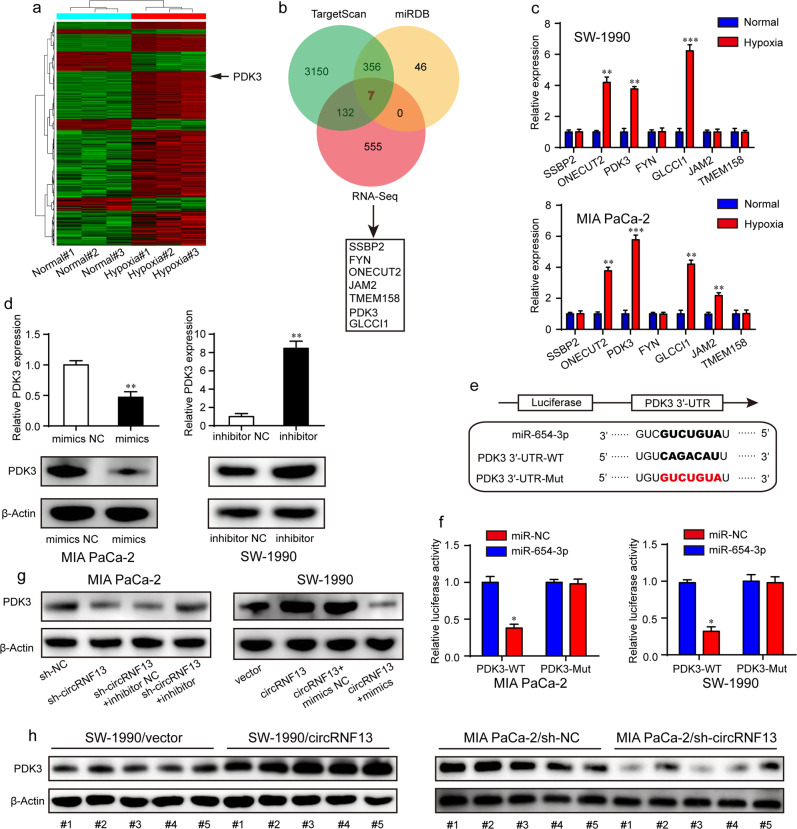
CircRNF13 modulates the miR-654-3p target PDK3. **a** Hierarchical clustering of significantly differentially expressed mRNAs in MIA PaCa-2 cells under normal and hypoxic conditions. **b** Schematic illustration showing the targets of miR-654-3p. **c** qRT–PCR was performed to measure gene expression under hypoxia. **d** PDK3 mRNA and protein levels after PC cells were transfected with miR-654-3p mimics or inhibitor. **e** Schematic illustration of wild-type and mutant reporter plasmids. **f** The luciferase activities in MIA PaCa-2 and SW-1990 cells transfected with miR-654-3p mimics or mimics NC. **g** The effects of circRNF13 and miR-654-3p on the expression of PDK3. **h** PDK3 levels in xenograft tumors removed from each group. Data represent at least three independent experiments and are presented as the means ± SD. *P < 0.05, **P < 0.01, ***P < 0.001.

Our previous study showed that miRNA exerts its biological function by binding to the 3′ untranslated region (3′-UTR) of mRNAs^20^. Candidate targets of miR-654-3p were first determined using miRDB and TargetScan prediction engines combined with mRNA profiling in the above PC cells cultured in hypoxic and normal conditions (Fig. 7a), in which the upregulated genes were selected. As listed in Fig. 7b, 7 genes were finally identified. Of these, GLCCI1, ONECUT2 and PDK3 exhibited significant overexpression under hypoxic conditions in SW-1990 and MIA PaCa-2 cells (Fig. 7c), whereas only PDK3 mRNA levels decreased or increased with the overexpression or knockdown of miR-654-3p (Fig. 7d). Thus, we selected PDK3 as a potential target of miR-654-3p for further study. In addition, PDK3 has recently been reported to play a critical role in tumor metabolism and is considered a potential therapeutic target for a variety of tumors^35,36^. To determine whether miR-654-3p regulated PDK3, we constructed a wild-type (WT) PDK3 3′-UTR luciferase reporter plasmid and a mutant reporter plasmid (Mut) in which these recognition sites were mutated (Fig. 7e). The results indicated that the luciferase activity was dramatically decreased after cotransfection of miR-654-3p mimics and wild-type PDK3 3′-UTR, while the luciferase activity of mutant PDK3 3'-UTR showed no difference between miR-654-3p mimics and the control group (Fig. 7f). Western blotting analysis demonstrated that miR-654-3p mimics decreased, whereas miR-654-3p inhibitor increased the level of PDK3 protein in PC cells (Fig. 7d). Additionally, circRNF13 overexpression significantly upregulated PDK3 protein expression, and miR-654-3p mimics eliminated this protein generation. In contrast, circRNF13 knockdown decreased PDK3 protein expression, and this effect could be rescued by the miR-654-3p inhibitor (Fig. 7g). As expected, SW-1990/circRNF13 xenograft tumors exhibited significantly higher levels of PDK3 protein, and PDK3 expression was decreased in MIA PaCa-2/sh-circRNF13 xenograft tumors (Fig. 7h). These results indicate that circRNF13 acts as a miR-654-3p sponge to increase target gene PDK3 expression.

### CircRNF13 accelerates PC progression through PDK3

**Fig. 8:**
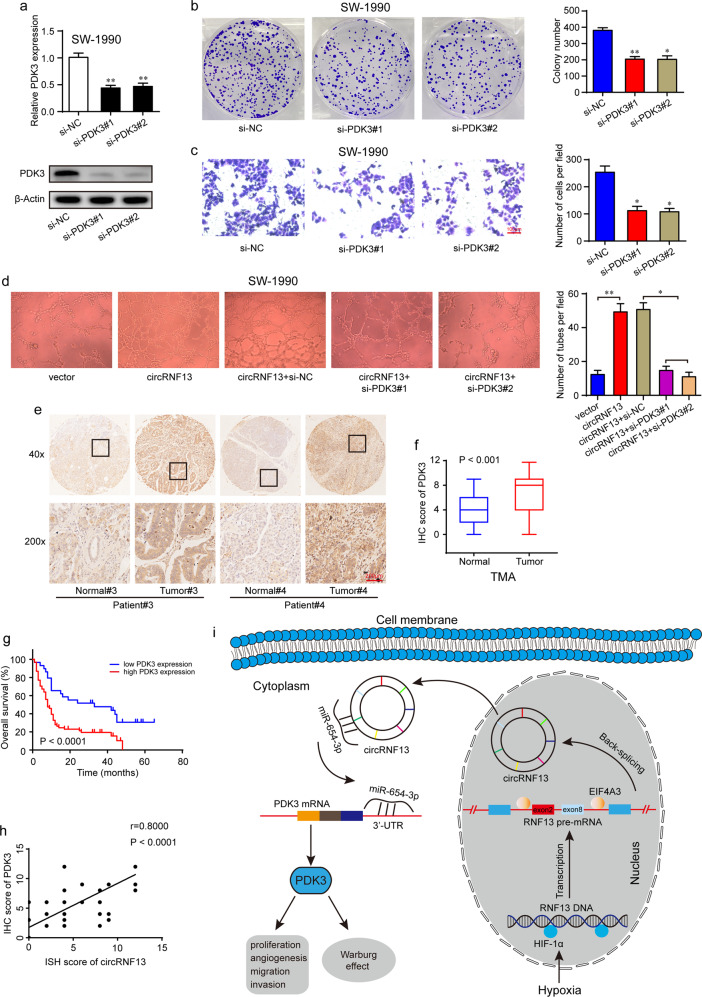
CircRNF13 promotes the progression of PC via PDK3. **a** The knockdown efficiency of PDK3 was verified at the mRNA and protein levels in SW-1990 cells by qRT–PCR and Western blotting assays. **b** Knockdown of PDK3 impaired colony formation ability in SW-1990 cells. **c** Knockdown of PDK3 impaired cell invasion ability in SW-1990 cells (scale bar: 100 μm). **d** Tube formation ability of HUVECs cultured in medium collected from circRNF13-overexpressing SW-1990 cells transfected with PDK3 siRNAs or their corresponding controls. **e** Representative IHC images of PDK3 expression in PC tissues and corresponding normal tissues (scale bar: 200 μm, magnification: top 40x, bottom 200x). **f** IHC scores of PDK3 in 90 cases of PC tissues with corresponding normal tissues. **g** Kaplan–Meier overall survival analysis of PDK3 expression in patients with PC (*N* = 90, *P* < 0.001). **h** Pearson correlation analysis of the positive correlation between circRNF13 and PDK3 expression (*r* = 0.800, *P* < 0.0001). **i** Graphical illustration demonstrating the circRNF13/miR-654-3p/PDK3 axis in pancreatic cancer. Data represent at least three independent experiments and are presented as the means ± SD. *P < 0.05, **P < 0.01.

To further explore the function of PDK3 in PC, two different siRNAs targeting PDK3 were designed, and the transfection efficiency was confirmed by qRT–PCR and Western blotting (Fig. 8a). PDK3 knockdown dramatically decreased cell proliferation (Fig. 8b), migration (Supplementary Fig. 7a), invasion (Fig. 8c) and tube formation ability (Supplementary Fig. 7b). A Seahorse stress test was also performed to detect the effect of PDK3 on glycolysis and oxidative phosphorylation. The results showed that the ECAR was decreased, while the OCR was increased after PDK3 knockdown in SW-1990 cells (Supplementary Fig. 7c). The above results indicated that PDK3 acted as a tumor oncogenic gene in pancreatic cancer. Furthermore, knockdown of PDK3 markedly suppressed circRNF13-induced PC cell proliferation (Supplementary Fig. 7d), migration (Supplementary Fig. 7e), invasion (Supplementary Fig. 7f) and tube formation (Fig. 8d). In addition, the PDK3 level in pancreatic cancer tissues was determined in the above pancreatic TMA. The IHC results showed that PDK3 was highly expressed in pancreatic cancer tissues compared with matched normal tissues (Fig. 8e, f). Further analysis showed that PDK3 levels were positively correlated with T stage (*P* = 0.004), N stage (*P* = 0.0007) and AJCC stage (*P* = 0.0042) (Supplementary Table 2 and Supplementary Fig. 7g). Survival analysis also showed that high expression of PDK3 predicted poor prognosis in PC patients (Fig. 8g). Pearson correlation analysis revealed a positive correlation between circRNF13 and PDK3 expression (*r* = 0.800, *P* < 0.0001) (Fig. 8h). These results further confirmed the regulatory effects of circRNF13 on PDK3.

## Discussion

In the past several decades, the role of noncoding RNAs in oncogenesis and cancer progression has become one of the major scientific discoveries. Among these, circRNAs have gained widespread attention in recent years due to their stability and wide dysregulation in various tumors^15,37^. Increasing evidence has suggested an important regulatory role of circRNAs in cancer biology. However, their roles in hypoxia have rarely been reported. In this study, we performed circRNA sequencing in PC cells cultured under hypoxic and normoxic conditions. We focused on a new circRNA termed circRNF13, which was dramatically upregulated under hypoxia. Further detection suggested that circRNF13 was overexpressed in pancreatic cancer tissues and associated with clinicopathological features and poor prognosis of pancreatic cancer patients.

Recent research has shown that the biogenesis of circRNAs is regulated by intronic complementary sequences and certain RNA binding proteins. Our current study demonstrated that circRNF13 is a hypoxia-responsive gene and is regulated by HIF-1α. The cardinal involvement of circRNA in hypoxia-associated metabolism has been studied recently. For example, circRNA-403658 was reported to be a hypoxia-responsive circRNA in bladder cancer. CircRNA-403658 silencing inhibited aerobic glycolysis and cell growth; thus, it may function as a potential therapeutic target in bladder cancer^38^. Another study in pancreatic cancer cells showed that circ-0000977, a hypoxia-inducible circRNA, could regulate the killing effect of NK cells through HIF1A and ADAM10^39^. Next, bioinformatic analysis revealed the EIF4A3 binding sites in the flanking intron sequences of RNF13 pre-mRNA. Subsequent RNA pulldown and RIP assays confirmed the enrichment of EIF4A3 in the downstream and upstream binding sites of the circRNF13 mRNA transcript. Although several studies have reported that EIF4A3 regulates circRNA formation, the specific regulatory mechanism still needs to be further explored. Perhaps there may be other RNA-binding proteins involved in circRNF13 biogenesis under hypoxia.

A growing number of studies have shown that circRNAs play diverse and vital biological roles in the progression of pancreatic cancer. In our study, gain- and loss-of-function experiments demonstrated that circRNF13 promoted the growth and metastasis of pancreatic cancer in vitro and in vivo. Moreover, circRNF13 was confirmed to play an important role in hypoxia-induced tumor progression and metabolic alterations. CircRNAs can act as miRNA sponges, thus counteracting the inhibitory effect of miRNA-mediated downstream targets, and this is the most reported underlying mechanism of circRNA function^40^. For instance, circTP63 promoted the expression of FOXM1 and its downstream molecules CENPA and CENPB by binding to miR-873-3p, exerting oncogenic potential in lung squamous cell carcinoma^41^. In hepatocellular carcinoma, silencing of circMTO1 promotes cell proliferation and invasion by sponging miR-9 to induce p21 expression^42^. In our study, we screened 5 potential miRNAs that could interact with circRNF13. Subsequently, RNA pulldown assays, dual-luciferase reporter assays and FISH assays verified that miR-654-3p was capable of binding to circRNF13. Then, the rescue experiments further confirmed that circRNF13 functions as a miRNA sponge for miR-654-3p. Furthermore, we combined bioinformatics software with a series of validating experiments to verify PDK3 as the downstream target gene of miR-654-3p.

Glucose metabolism from OXPHOS to glycolysis is a unique metabolic characteristic of cancer cells^29^. It is generally thought that pyruvate kinase, phosphofructokinase (PFK) and hexokinase (HK) are three rate-controlling enzymes in these metabolic alterations^43^. Among them, the PDK family, including PDK1, PDK2, PDK3 and PDK4, regulates the balance of glycolytic and OXPHOS metabolism through pyruvate dehydrogenase (PDH) and plays a critical role in tumor metabolism^44^. PDK3 drew our attention because of its increasing importance in cancer metabolism. A new study suggested that PDK3 expression was increased in chemoresistant gastric cells. It forms a positive feedback loop with the transcription factor HSF1 to drive glycolysis^45^. Moreover, the oncogenic role of PDK3 was also discovered in other cancers, such as colon cancer^46^, lung cancer^47^ and acute myeloid leukemia^48^. Nevertheless, the function of PDK3 in pancreatic cancer has not been determined. In our study, we found that circRNF13 combined with miR-654-3p promoted the expression of PDK3. Functional experiments showed that PDK3 played an oncogenic role in PC cells. Subsequent rescue experiments indicated that PDK3 knockdown reversed the circRNF13-induced biological behaviors of PC cells and angiogenesis. Then, we further detected the level of PDK3 in PC tissues and revealed its positive correlation with circRNF13, indicating the clinical significance of the circRNF13/miR-654-3p/PDK3 axis during PC progression.

This was the first study to reveal circRNA expression profiles in hypoxia-induced PC cells. We identified circRNF13 in our study, the biological function of which has not been reported in PC. Our findings may provide clues for research on hypoxia and circRNAs in other cancer types. However, several limitations in our results need to be taken into consideration. First, circRNF13 was proven to be highly expressed in PC tissues and associated with poor prognosis in pancreatic cancer patients, but whether circRNF13 can be detected in patient serum needs further investigation. Second, we proved that hypoxia-induced circRNF13 promoted PC cell proliferation, migration and invasion in vitro. Importantly, we also demonstrated that hypoxia-induced cell migration and invasion could be eliminated after circRNF13 knockdown in vitro. The above results showed that circRNF13 is critical for hypoxia responses in PC cellular biological processes. In vivo, we utilized xenograft and metastatic models to prove the role of circRNF13 in promoting PC growth and metastasis. The KPC (Pdx1-Cre; K-Ras^G12D/+^; p53^R172H/+^) model is a well-validated, clinically relevant model of PC, in which tumors arise spontaneously with the development of defined histopathological stages of progression that mirror human disease^49^. In future studies, the function of circRNF13 and its relationship with the hypoxic tumor microenvironment in PC could be investigated in the KPC model. Third, circRNAs have multiple functions, including adsorbing miRNAs, which are one of the most extensively studied, coding potentials and are involved in protein translation^50^. In our research, we verified the ability of circRNF13 to bind to miR-654-3p, but whether circRNF13 promotes the malignant process of PC through other functions needs to be further explored. Last, it has been reported that PDK3 is induced by hypoxia and directly regulated by HIF-1α at the transcriptional level^51^, while another study demonstrated that PDK3 expression was not reduced after depleting HIF-1α^45^. Our finding is the first to reveal the regulation of PDK3 by miR-654-3p, but whether PDK3 is directly regulated by HIF-1α requires further study.

In summary, our research identified a novel hypoxia-responsive circRNF13 in PC cells. The level of circRNF13 was found to be upregulated in PC tissues and correlated with poor prognosis in PC patients. More importantly, we revealed that HIF-1α and EIF4A3 mediate the biogenesis of circRNF13. Functional experiments demonstrated an oncogenic role of circRNF13 in PC development and glucose metabolism. Mechanistically, circRNF13 promoted PC tumorigenesis and metastasis by regulating the miR-654-3p/PDK3 axis (Fig. 8i). Therefore, targeting the circRNF13/miR-654-3p/PDK3 axis might provide new perspectives in the diagnosis and treatment of PC.

## Supplementary information

Supplementary figures and tables
