# Supplementary material for: Hypoxia-induced circRNF13 promotes the progression and glycolysis of pancreatic cancer
Source: Exp Mol Med. 2022 Nov 11;54(11):1940–54. doi: 10.1038/s12276-022-00877-y (PMC9723180; doi:10.1038/s12276-022-00877-y)
Supplement: Supplementary file 1 — Supplementary figures and tables [file 12276_2022_877_MOESM1_ESM.pdf]

Supplementary Fig. 1

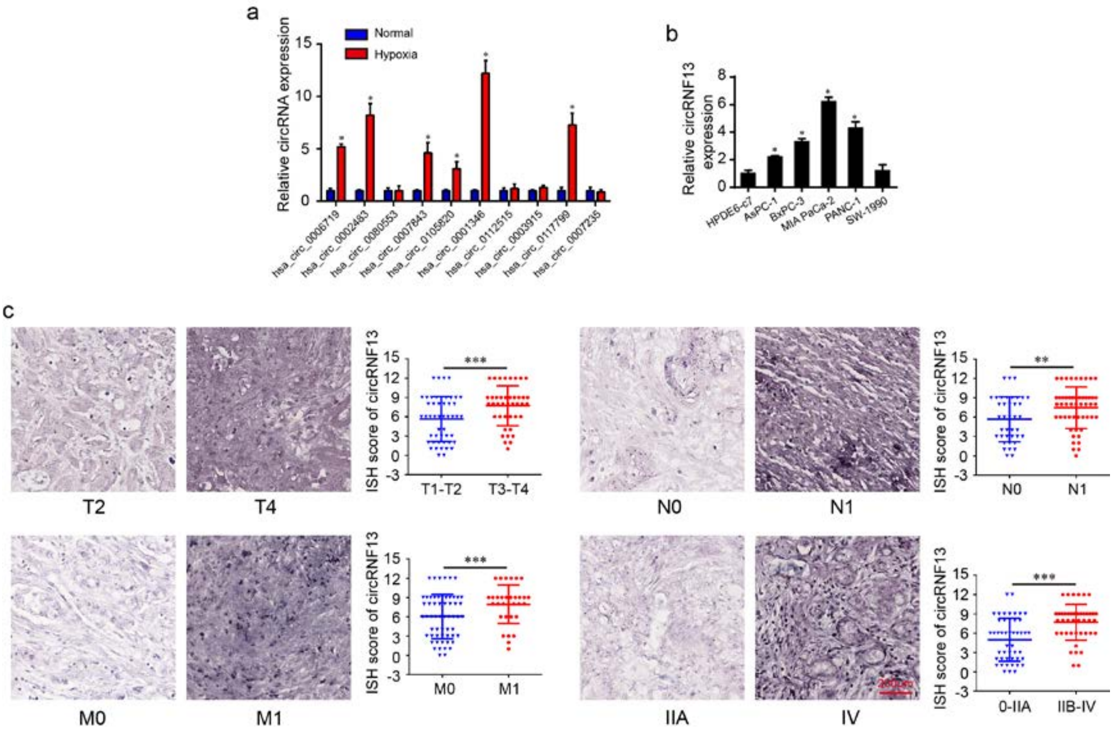

Expression profiles of circRNA in hypoxia-induced PC cells.

a QRT-PCR confirmed the expression of top 10 upregulated circRNAs under hypoxia in MIA PaCa-2 cells. **b** QRT-PCR analysis of the relative expression of circRNF13 in PC cells compared with normal pancreatic ductal epithelial cells (HPDE6-c7). **c** IHC analysis of circRNF13 expression in PC tissues of T, N, M and AJCC stages. Data represent at least three independent experiments and present as means ± SD. *P < 0.05, **P < 0.01.

Supplementary Fig. 2

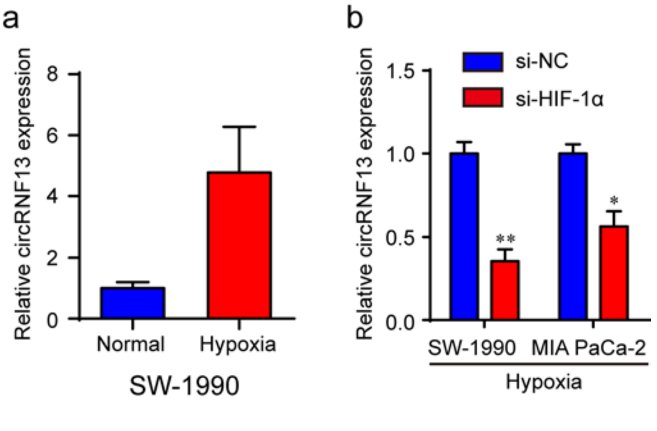

CircRNF13 is hypoxia-inducible.

a QRT-PCR analysis of circRNF13 level in SW-1990 cells under hypoxia. **b** QRT-PCR analysis of circRNF13 level after HIF-1α knockdown. Data represent at least three independent experiments and present as means ± SD. *P < 0.05, **P < 0.01.

Supplementary Fig. 3

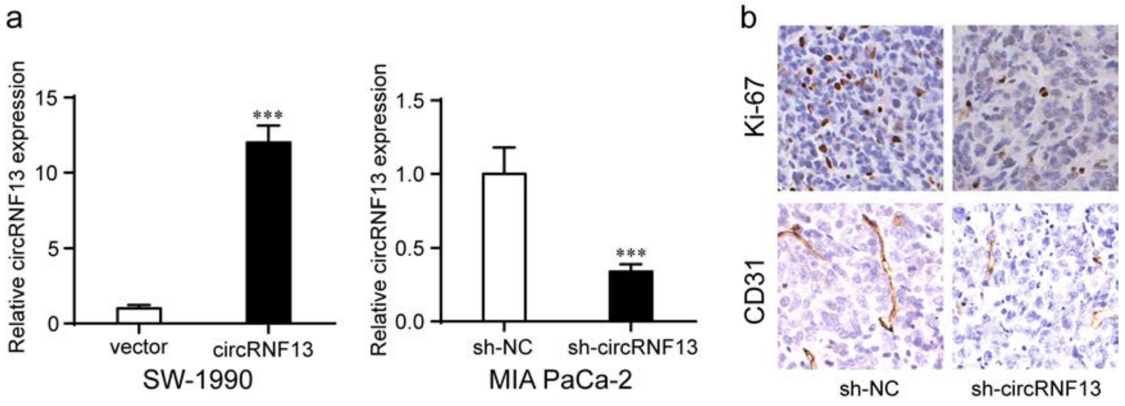

a CircRNF13 overexpression and knockdown efficiency was determined by qRT-PCR. **b** Ki-67 and CD31 expression in tumor tissues of circRNF13 knockdown group and control group (scale bar: 100 μm, magnification: 200 x). Data represent at least three independent experiments and present as means ± SD. ***P < 0.001.

Supplementary Fig. 4

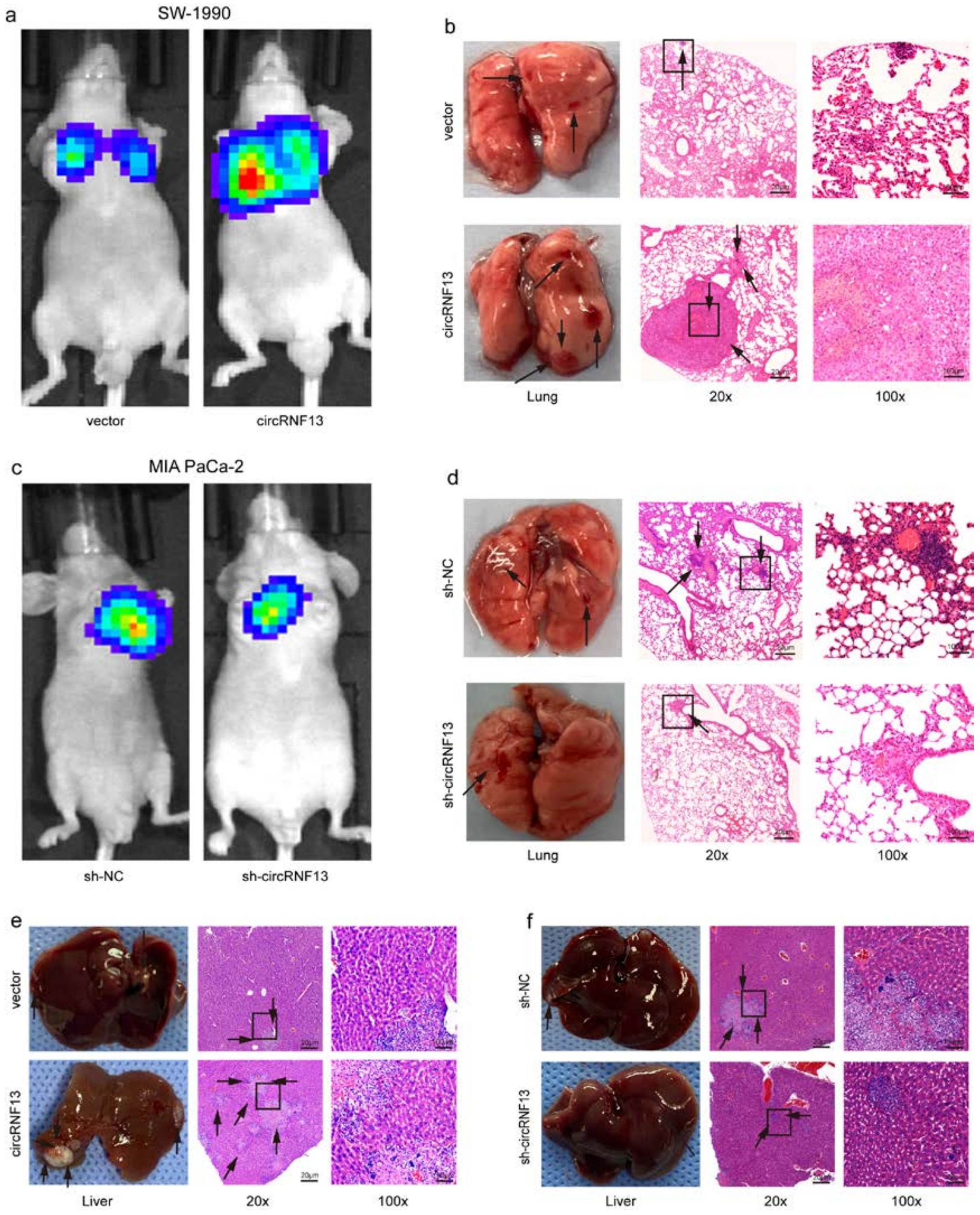

CircRNF13 promotes PC metastasis *in vivo*.

a, c Representative bioluminescence images of mice 8 weeks after tail vein injection of SW-1990/circRNF13 and MIA PaCa-2/sh-circRNF13 cells. **b, d** Representative images of metastatic nodes in the lungs and HE staining of the lung tissues of respective groups. **e, f** Representative liver images and HE staining of the liver tissues of respective groups. Data represent at least three

independent experiments and present as means ± SD. *P < 0.05, **P < 0.01.

Supplementary Fig. 5

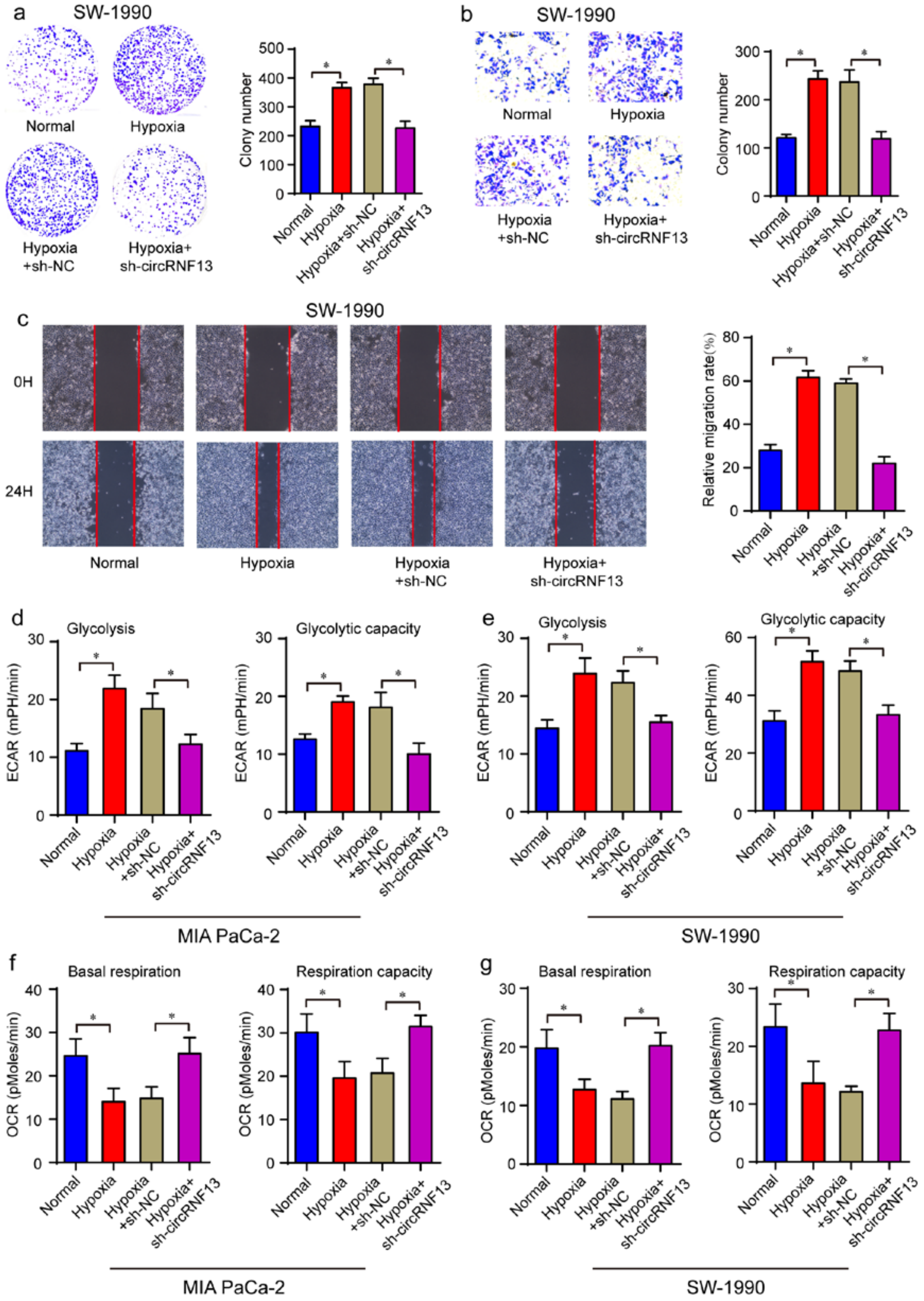

CircRNF13 silence suppresses the tumor-promoting effects of

hypoxia in PC cells.

a Colony formation assay was used to analyze the effect of circRNF13 silence on the hypoxia induced cell proliferation. **b, c** Transwell and wound healing assays were conducted to detect the migration and invasion abilities. **d, e** ECAR after glucose (GLU) treatment indicated the glycolysis rate. ECAR after oligomycin (OLI) treatment indicated glycolytic capacity. **f, g** OCR before oligomycin treatment indicated basal respiration. OCR after FCCP treatment indicated maximum respiration capacity. Data represent at least three independent experiments and present as means ± SD. *P < 0.05, **P < 0.01.

Supplementary Fig. 6

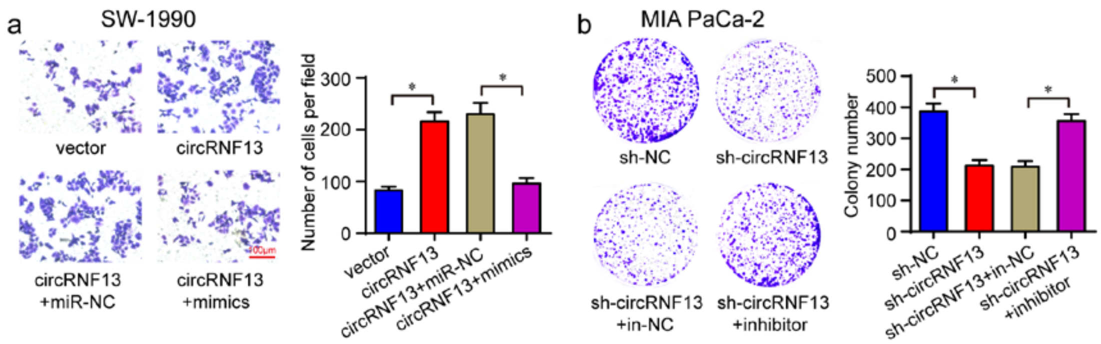

CircRNF13 promotes PC cell progression by sponging miR-654-3p.

a Cell invasion ability was detected in circRNF13 overexpressing SW-1990 cells transfected with miR-654-3p mimics or mimics NC. **b** Cell proliferation ability was determined in circRNF13 knockdown MIA PaCa-2 cells transfected with miR-654-3p inhibitor or inhibitor NC. Data represent at least three independent experiments and present as means ± SD. *P < 0.05, **P < 0.01.

Supplementary Fig. 7

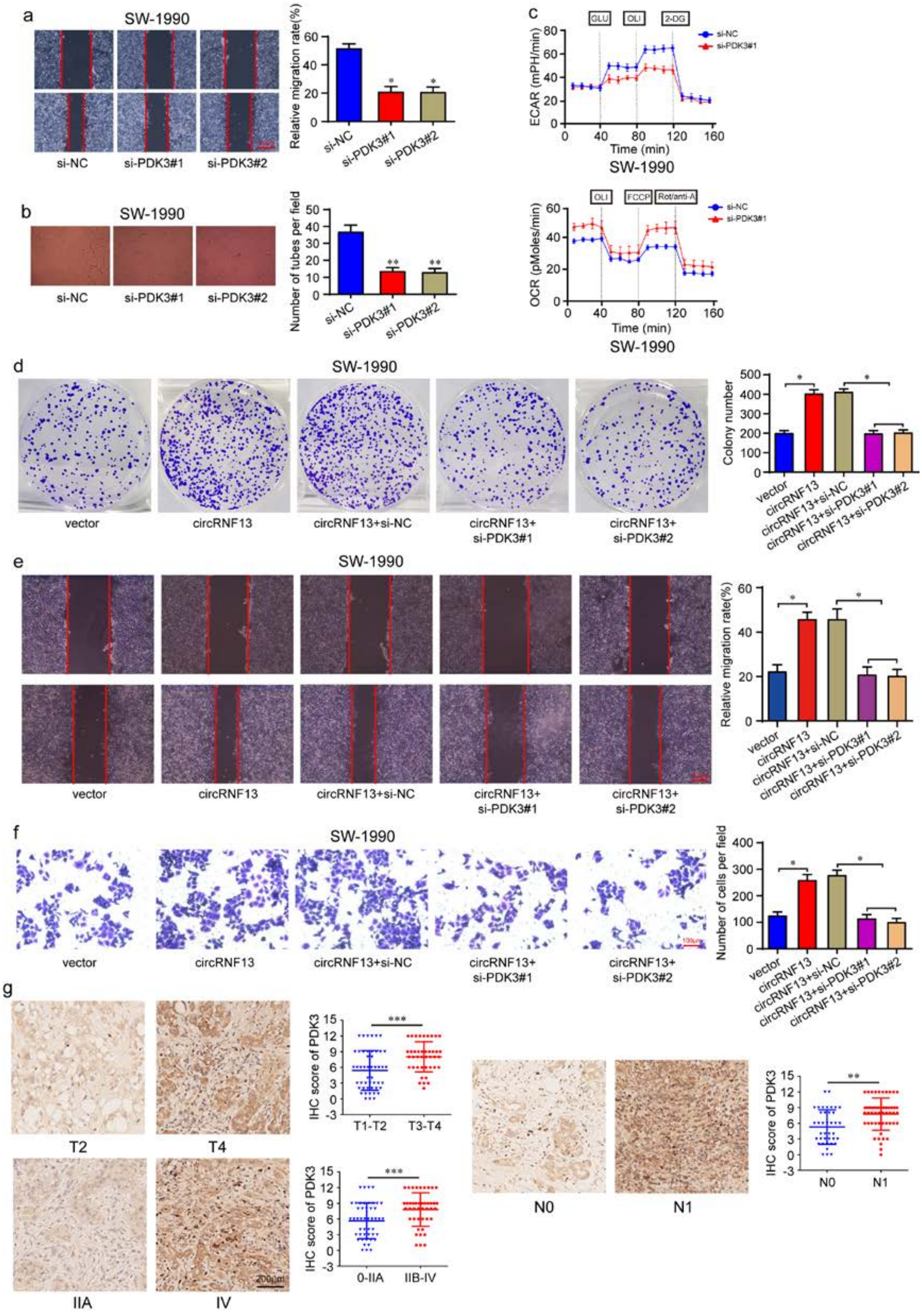

CircRNF13 accelerates the PC malignant process through PDK3.

a Knockdown of PDK3 impaired cell migration ability in SW-1990 cells (scale

bar: 20 μm). **b** Knockdown of PDK3 impaired tube formation ability. **c** ECAR and OCR in SW-1990 cells were measured by the Seahorse XF96 extracellular Flux analyzer. **d** Cell colony formation ability of circRNF13-overexpressing SW-1990 cells transfected with PDK3 siRNAs or their corresponding controls. **e** Representative images of the cell migration ability of circRNF13-overexpressing SW-1990 cells transfected with PDK3 siRNAs or their corresponding control (scale bar: 20 μm). **f** Cell invasion ability of circRNF13-overexpressing SW-1990 cells transfected with PDK3 siRNAs or their corresponding control (scale bar: 100 μm). **g** Representative IHC images of PDK3 expression in T2 *vs* T4, N0 *vs* N1 and AJJC IIA *vs* IV. Data represent at least three independent experiments and present as means ± SD. *P < 0.05, **P < 0.01.

Supplementary Table 1. The relationship of circRNF13 and clinicopathologic parameters in 90 PC patients

Variables	No. (n=90)	circRNF13 expression high (n=62)	low (n=28)	P
Gender				
Male	58	38	20	0.352
Female	32	24	8	
Age (years)				
≤60	40	25	15	0.242
>60	50	37	13	
Pathologic grade				
Low	33	22	11	0.729
Middle-High	57	40	17	
T stage				
T1-T2	51	28	23	**0.001**
T3-T4	39	34	5	
N stage				
N0	43	21	22	**< 0.0001**
N1-N2	47	41	6	
M stage				
M0	73	46	27	**0.01**
M1	17	16	1	
AJCC stage				
0-IIA	36	15	21	**< 0.0001**
IIB-IV	54	47	7	

Supplementary Table 2. The relationship of PDK3 and clinicopathologic parameters in 90 PC patients

Variables	No. (n=90)	PDK3 expression high (n=67)	PDK3 expression low (n=23)	P
Gender				
Male	58	40	18	0.108
Female	32	27	5	
Age (years)				
≤60	40	28	12	0.387
>60	50	39	11	
Pathologic grade				
Low	33	22	11	0.198
Middle-High	57	45	12	
T stage				
T1-T2	51	32	19	**0.004**
T3-T4	39	35	4	
N stage				
N0	43	25	18	**0.0007**
N1-N2	47	42	5	
M stage				
M0	73	52	21	0.2193
M1	17	15	2	
AJCC stage				
0-IIA	36	21	15	**0.0042**
IIB-IV	54	46	8	
